# Effect of thermocycling on surface topography and fracture toughness of milled and additively manufactured denture base materials: an in-vitro study

**DOI:** 10.1186/s12903-024-03991-7

**Published:** 2024-02-23

**Authors:** Mohamed M. Abdul-Monem, Kenda I. Hanno

**Affiliations:** 1 Department of Dental Biomaterials, Faculty of Dentistry, University of Alexandria, Alexandria, Egypt; 2 Division of Dental Biomaterials, Department of Prosthodontics, Faculty of Dentistry, Alamein International University, Alamein, Egypt; 3 Department of Prosthodontics, Faculty of Dentistry, University of Alexandria, Alexandria, Egypt

**Keywords:** CAD-CAM, Milled, 3D-printed, Thermocycling, Surface roughness, Fracture toughness, Hardness, Sorption, Solubility

## Abstract

**Background:**

Studies investigating thermocycling effect on surface topography and fracture toughness of resins used in digitally manufactured denture bases are few. The study aimed to assess the impact of thermocycling on surface topography and fracture toughness of materials used for digitally manufactured denture bases.

**Methods:**

Water sorption, solubility, hardness, surface roughness, and fracture toughness of both three-dimensional (3D)-printed and computer-aided design, computer-aided manufacturing (CAD-CAM) milled specimens (*n* = 50) were assessed both prior to and following 2000 thermocycles, simulating 2 years of clinical aging. Surface hardness (*n* = 10) was measured using a Vickers hardness testing machine, surface roughness (*n* = 10) was determined by a contact profilometer, and fracture toughness (*n* = 20) was measured using the 3-point bend test, then studying the fractured surfaces was done via a scanning electron microscope (SEM). Prior to and following thermocycling, water sorption and solubility (*n* = 10) were assessed. Normally distributed data was tested using two-way repeated ANOVA and two-way ANOVA, while Mann Whitney U test and the Wilcoxon signed ranks test were used to analyze data that was not normally distributed (α < 0.05).

**Results:**

Following thermocycling, Vickers hardness and fracture toughness of both groups declined, with a significant reduction in values of the 3D-printed resin (*P* < .001). The 3D-printed denture base resins had a rougher surface following thermocycling with a significant difference (*P* < .001). The sorption and solubility of water of both materials were not affected by thermocycling.

**Conclusions:**

Before and after thermocycling, milled specimens had lower surface roughness and a greater degree of hardness and fracture toughness than 3D-printed specimens. Thermocycling lowered hardness and fracture toughness, and increased surface roughness in both groups, but had no effect on water sorption and solubility.

**Supplementary Information:**

The online version contains supplementary material available at 10.1186/s12903-024-03991-7.

## Background

Complete dentures (CDs) have been made using the traditional compression mold or the flask-pack-press for more than 50 years. Polymethylmethacrylate (PMMA) resin and heat polymerization have been used to produce CDs [1].

The advancement of CAD-CAM processes for CDs, however, has caused a notable revolution in this protocol in recent years [2]. In contrast to conventional methods, CAD-CAM dentures may be constructed without the need for time-consuming labor-intensive processes [3–6]. Digital methods have the advantage of faster denture fabrication and fewer stages in the process, which can lower the likelihood of errors [7, 8].

The process of creating digital dentures was first established as a subtractive technique in which the dentures were manufactured from pre-polymerized resin blocks. Research has concluded that milled resins exhibit higher surface and mechanical properties [4, 9–12], reduced microbial colonization [13, 14], a decreased leach rate of residual monomer [15], and comparable color stability [16–18], in comparison to compression molded resins, because controlled conditions and high pressure were used during manufacturing of pre-polymerized blocks.

Later, the additive approach was introduced, in which digital technology was used to manufacture dentures layer by layer utilizing 3D printing technology and liquid resins polymerized using ultraviolet light [19]. Dentistry has made use of a variety of 3D printers and materials, such as fused deposition modeling (FDM), thermal inkjet (TIJ), and selective laser sintering (SLS) [20]. This method is more cost-effective because it allows for the simultaneous production of several products and eliminates the need for rotary tool wear and raw material waste [21, 22].

It is crucial to evaluate the surface qualities and mechanical characteristics of CAD-CAM materials to guarantee their success [23–27]. The wettability, hardness, and surface properties of acrylic resins may have an impact on plaque buildup [28, 29]. Dentures are constantly exposed to temperature fluctuations brought on by food and beverages [30, 31]. The characteristics of dental materials may be negatively impacted by these temperature fluctuations, especially if they are experienced frequently [30]. As a result, it is critical to evaluate denture base material performance in settings that mimic the intraoral environment [32].

Due to temperature variations brought on by ingested foods and beverages, the intraoral environment is a thermally dynamic medium [24]. The oral cavity’s temperature fluctuates 20 to 50 times a day, or 10,000 times a year on average [32] A recognized technique for subjecting dental materials to water baths with varying temperatures and simulating intraoral temperature changes is thermal cycling at 5° to 55 °C for 30 s [30, 33]. The surfaces of dentures may degrade as a result of thermal stresses brought on by these temperature variations [32, 34].

Research has shown that CAD-CAM milled resins have improved properties compared with 3D-printed resins such as higher fracture toughness, lower water sorption [35–37], and higher flexural modulus [38], while another study showed higher fracture toughness for 3D-printed resins [39]. Additional research [40–43] focused on the color stability and roughness of the surface of denture base resins. There is insufficient data on how thermal stresses affect the surface characteristics and fracture toughness of digitally manufactured resins used for denture bases, thus it is important to test the effect of thermal stresses to have a better prediction of which type will have a greater resistance to thermal changes and is better suited for long term prostheses clinically.

The aim of our study is to evaluate the effect of thermal cycling on Vickers hardness, surface roughness, fracture toughness, sorption of water, and solubility of denture base materials that have been CAD-CAM milled and 3D-printed. The null hypothesis was that thermocycling would have no effect on the tested properties of 3D-printed and CAD-CAM milled denture base materials.

## Methods

The Committee of Research Ethics in Alexandria University, Faculty of Dentistry (IORG 0008839) has approved the research prior to any research-related activities.

Two groups (*n* = 50) were investigated in this study, 3D-printed resin (Denture base LP; Formlabs) and prepolymerized blanks (M-PM; Merz Dental GmbH), (Table 1). The estimated sample size was 100 specimens, based upon the assumptions of a 95% level of confidence and an 80% study power [32, 44]. A computer program (G*power 3.0.10; Heinrich Heine University Düsseldorf) [45] and Rosner technique [46] was used to calculate the sample size.

**Table 1:** Composition of 3D printing resin and prepolymerized blanks for CAD-CAM milling

Material	Brand Name	Composition
3D printing resin	Denture Base Resin LP Formlabs Inc., MA, USA	• 55–75% w/w urethane dimethacrylate • 15–25% w/w methacrylate monomers • < 0.9% w/w phenyl bis (2,4,6- trimethylbenzoyl)-phosphine oxide
Prepolymerized blanks for CAD-CAM milling	M-PM, Merz Dental GmbH, Lütjenburg, Germany	• Pre-polymerized PMMA: >98% • Methyl 2-methylprop-2-enoate • Methyl2-methylpropenoate • Methyl methacrylate: <1% • Dibenzoyl peroxide. • Benzoyl peroxide: <1%

For each material, Vickers hardness (*n* = 10), surface roughness (*n* = 10), fracture toughness(*n* = 20), solubility, and sorption (*n* = 10) were evaluated before and after thermocycling. The specimens were created using a software program (Autodesk Meshmixer; Autodesk Inc.) and stored as standard tessellation language (STL) files.

### Specimens’ dimensions

Specimens for Vickers hardness with measurements of 15 × 10 × 2.5 mm were fabricated in accordance with Organization for International Standardization (ISO) 20795-1:2013 [47]. For the surface roughness test, the specimens were disk shaped with dimensions of 10 × 2 mm [18]. In order to test for fracture toughness, specimens with measurements of 39 × 8 × 4 mm dimensions and a 3.2 mm precrack were manufactured in accordance with ISO 20795-1 [47]. Circular specimens with 15 × 2 mm size were fabricated to test the solubility and sorption of materials in water [48].

### Specimens’ fabrication

**Fig. 1:**
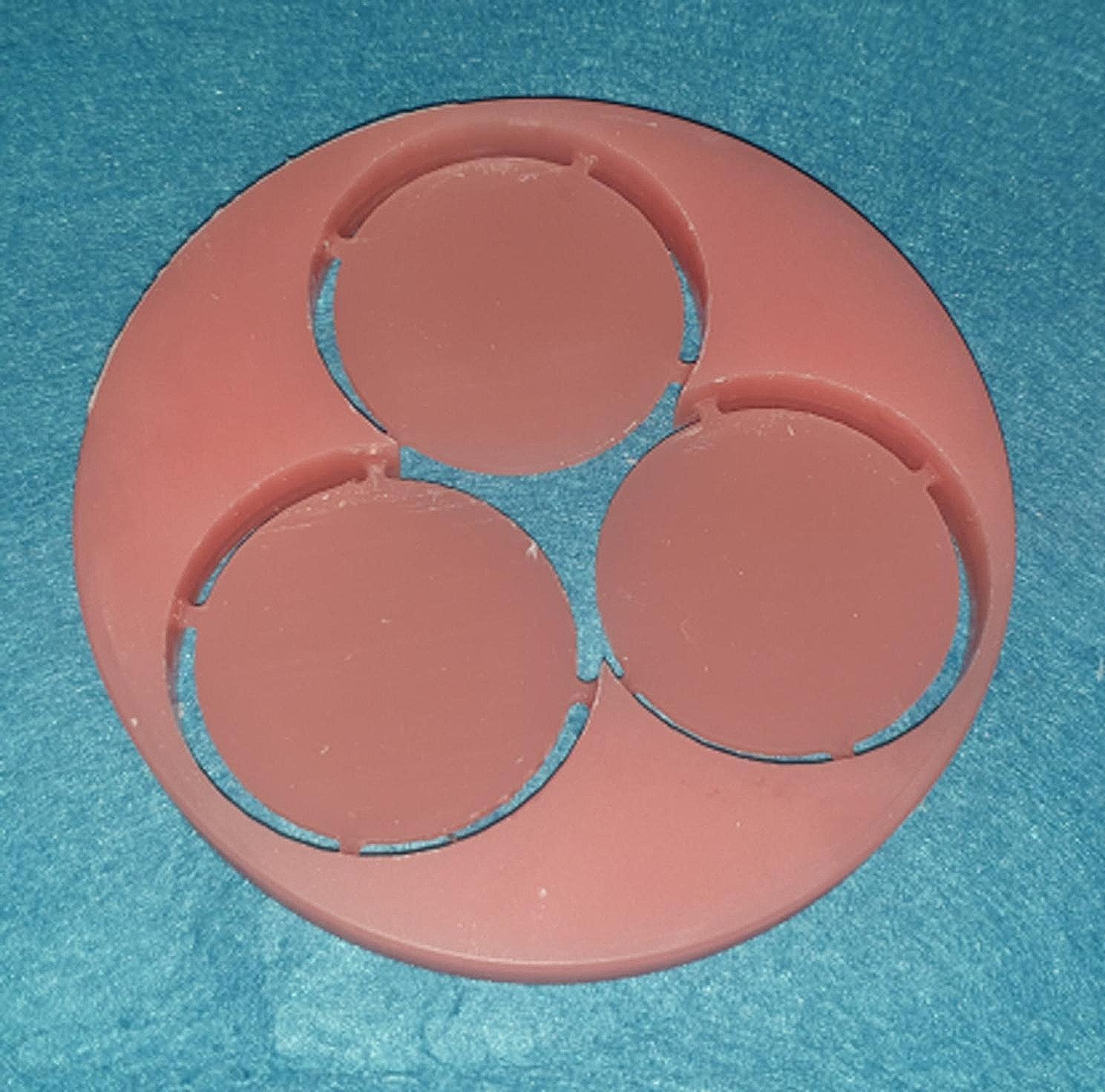
CAD-CAM milled specimens

**Fig. 2:**
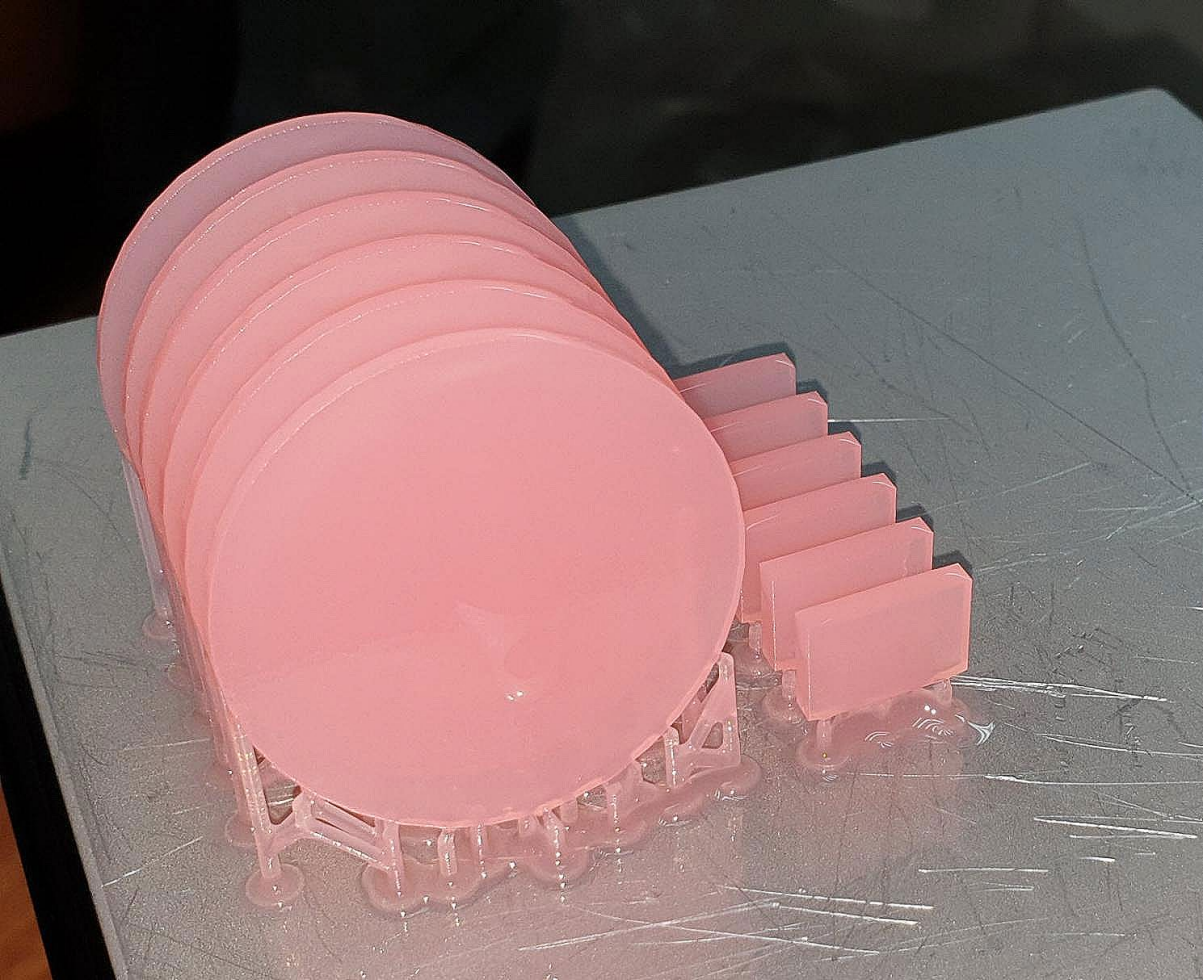
3D-printed specimens

In the CAD-CAM milled group, the specimens were milled to the specified dimensions (Fig. 1) from PMMA-based blanks using a computer numeric controlled (CNC) milling machine (Ceramill motion 2; AmannGirrbach). The specimens made by 3D printing (Fig. 2) were constructed by sending the files to a 3D printing machine (Form 3; Formlabs). Biocompatible denture base resin (Denture base OP; Formlabs) was used for printing at an angle of 45-degrees to the printing platform with 50 μm thickness of each layer [10], and the software program used for the printer (PreForm; Formlabs) was used to add supporting structures. Cleaning of the specimens was done using 99% isopropyl alcohol for 5 min in an ultrasonic bath (Sonorex RK; Bandelin) to remove any uncured or excess resin according to instructions stated by the manufacturer. The specimens were then submerged in glycerin and polymerized at 80 °C for 30 min using ultraviolet light in a postpolymerization lightbox (Form Cure; Formlabs) [35].

Silicon disks (CarbiMet; Buehler Ltd) were used to finish the specimens. A slurry of pumice (Pumice medium; Industrial plaster Ltd) with a lathe brush and a polishing paste (Universal polishing paste; Renfert GmbH) applied at a speed of 500 rpm [49] for 90 s, were used to polish the specimens. A single experienced operator completed the finishing and polishing procedures. The polishing of specimens was done according to manufacturer’s instructions, and this is recommended clinically to achieve a smooth denture surface.

### Thermal cycling

In accordance with ISO 20795-1 [47], the samples were kept in distilled water at 37 °C for 50 h (baseline). Half of the specimens then underwent 2000 cycles of thermal aging in a thermocycler (THE-1100; SD Mechatronik) to simulate the clinical aging of denture base materials for 2 years, with immersion times of 30 s in water that was 5 °C/55°C and dwell times of 10 s [50].

### Vickers hardness test

**Fig. 3:**
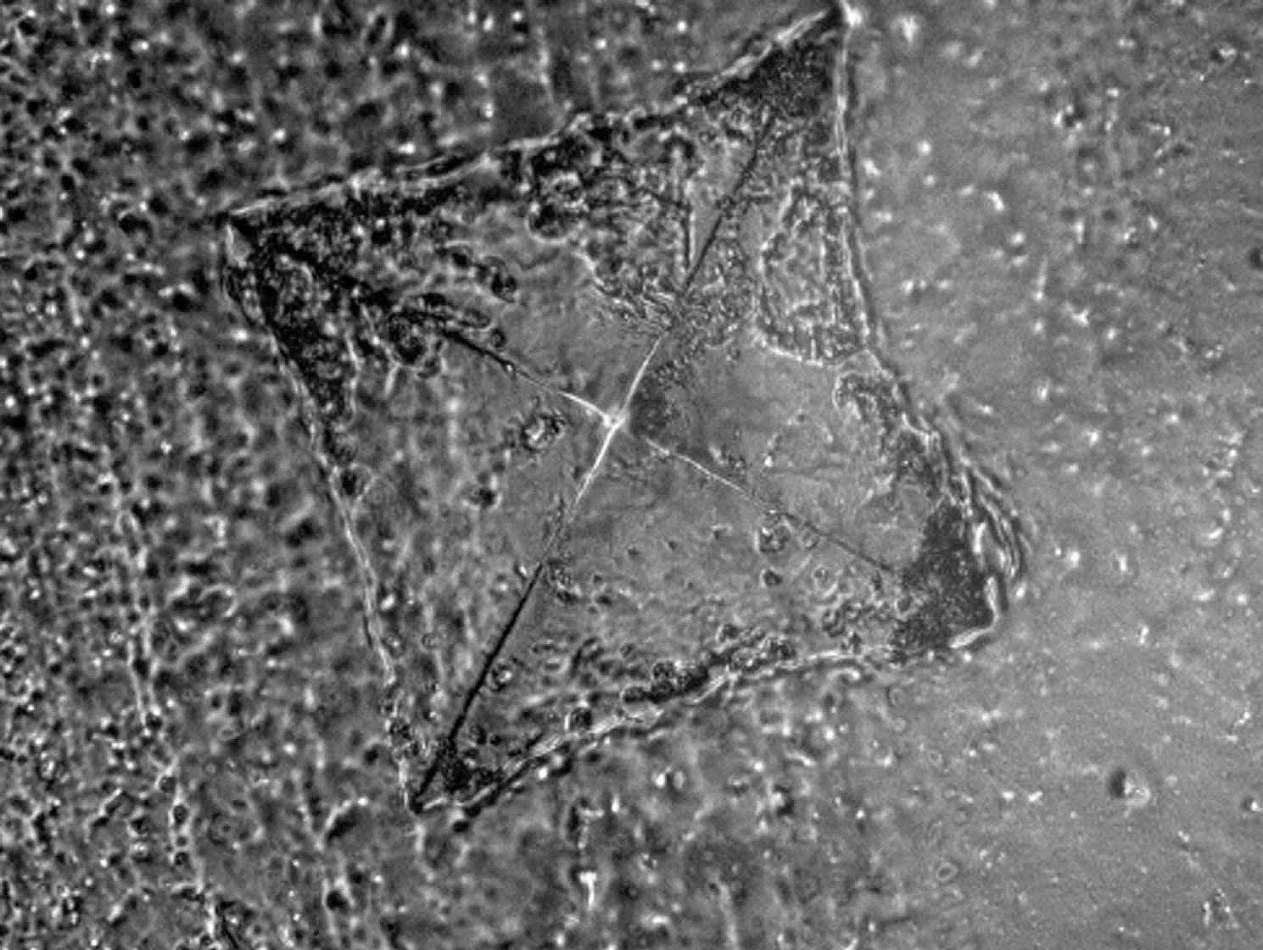
Vickers hardness Indentation in specimens showing a square with two diagonals

All the tested properties were assessed preceding and following thermocycling. Vickers hardness testing machine (FM-700, Future Tech Corp) was used to measure the hardness of the specimens (*n* = 10 per group). A diamond-shaped indenter was used to apply 50 g with a dwell period of 30 s for each specimen, 3 indentations were made, and the average was obtained [51]. The diagonals of the diamond-shaped indentations (Fig. 3) were measured to the closest 0.1 μm. The Vickers hardness number (VHN) was calculated using the formula: VHN = 0.185 L/d^2^, where L stands for load (N) and d for mean length of the diagonal (µm) [28].

### Surface roughness measurement

The contact profilometer (Surftest SV-3100, Mitutoyo Corp) was utilized to measure the specimens’ surface roughness (Ra) with a stylus speed of 1 mm/second and an endpoint of 0.8 mm (*n* = 10 per group). Three readings were obtained for each specimen, and the average was determined in micrometers (µm) [10, 50].

### Fracture toughness test

The fracture toughness test was conducted (*n* = 20 for each group) using the 3-point bend method using an all-purpose testing machine (Omnitest; Macmesin) with a 32 mm wide span and a rate of displacement of 1 mm/minute before thermocycling [47]. The test was conducted on 10 samples before thermocycling, and 10 other samples after thermocycling, because the specimens fracture during the test. Using the following equations [52], the fracture toughness (KIc) was calculated from the recorded highest force:

$${K}_{\begin{array}{c}Ic\\ \end{array}}=\frac{f\left(x\right).{F}_{max}. {l}_{t}}{{b}_{t}. {h}_{t}^{\frac{3}{2}}}.\surd {10}^{-3}$$

$$f\left(x\right)=\frac{{3x}^{\frac{1}{2}} [1.99-x\left(1-x\right)(2.15-3.93x+2.7{x}^{2}]}{\left[2\left(1+2x\right){\left(1-x\right)}^{\frac{3}{2}} \right]}, x=\frac{a}{{h}_{t}}$$,

where *Fmax* is the specimen maximum applied force expressed in Newtons, *a* represents the precrack length expressed in mm, *ht* denotes the height of specimen expressed in mm, *bt* denotes the width of the specimen mm, and *lt* represents the width of the span in mm. To assess the cracked surface utilizing a SEM (JSM 200 IT, JOEL) at magnification levels (×100) and (×300), specimens from the 2 groups were sputtered with gold.

### Water sorption and solubility test

Circular specimens (*n* = 10 per group) were dehydrated using silica gel at 37 °C until a consistent mass was observed for water solubility and sorption. The specimen weights were determined utilizing an analytical scale (AS 220.R2, RADWAG) with a precision of 0.1-mg precision. By employing a digital micrometer (Digimatic, Mitutoyo), the dimensions of each specimen were computed by taking the average of the 3 measurements taken on each side while the specimens’ mass remained fixed (m1) [53].

Specimens were weighed again (m2) following 7 days of submersion inside water at a temperature of 37 °C (baseline). Following 2000 thermocycles to assess the sorption with time, specimens were weighed (m3). Once an unaltered mass (m4) was attained, the specimens were once more dried in the desiccator. In line with ISO 4049 [54], calculating solubility (Wsl) and sorption (Wsp) involved use of the following formula:

Wsp 7 days = $\frac{m2-m1}{V}$ Wsp thermocycling = $\frac{m3-m1}{V}$ Wsl = $\frac{m4-m1}{V}$, where m is the dry specimen mass in mg before being submerged in water, m2 is the specimen mass following 7 days immersed in water and is measured in mg, m3 is the specimen mass following thermocycling in mg, m4 is the specimen mass following the 2nd drying in mg, and V is the specimen volume in mm^3^.

Q-Q plots and the Shapiro-Wilk test were used to verify the normality of the variables. Since the values for surface roughness, Vickers hardness, and fracture toughness were all normally distributed, the means, standard deviations, and 95% confidence intervals (CI) were used to present the data. However, sorption of water and solubility showed non normal distribution hence it was presented mainly using median, interquartile range (IQR), and minimum and maximum values. Percent change in all parameters was calculated based on the formula: [(values after thermocycling – values before thermocycling) / values before thermocycling] x 100. To clarify how denture material and thermocycling, as well as their combined effects, affect fracture toughness, a two-way ANOVA was used, while 2 -way repeated measures ANOVA were performed to analyze Vickers hardness and surface roughness. The Wilcoxon Sign Rank test was used to evaluate variations in water sorption within groups, and the Mann Whitney U test was used for comparing water sorption, solubility, and percent change. *P* value < 0.05 was used as the significance level, and all tests were two-tailed. IBM SPSS version 23, Armonk, NY, USA, was used to analyze the data.

## Results

**Fig. 4:**
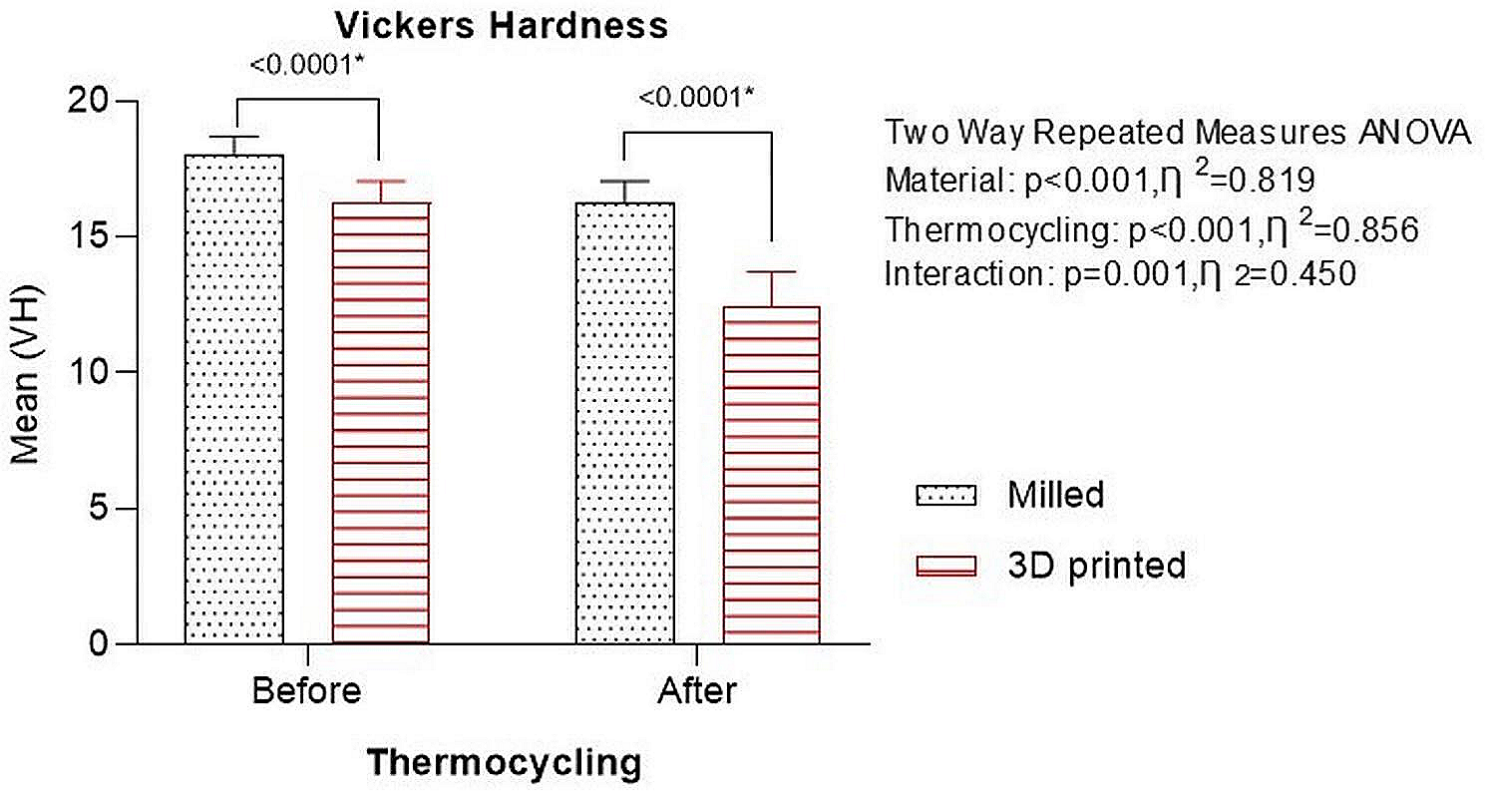
Graph showing Vickers hardness of CAD-CAM milled and 3D-printed denture base resins before and after thermocycling

Results revealed that Vickers hardness of CAD-CAM milled denture base material was higher compared with 3D-printed resins before (18.02 ± 0.67 and 16.26 ± 0.79, respectively) and after thermal cycling (16.26 ± 0.79 and 12.42 ± 1.30, respectively). Both groups showed a decline in Vickers hardness following thermocycling (Fig. 4). The two-way repeated ANOVA displayed a significant effect of the denture base material (*P* < .001), thermocycling (*P* < .001), and a significant interaction of both factors affecting Vickers hardness (*P* = .001).

**Fig. 5:**
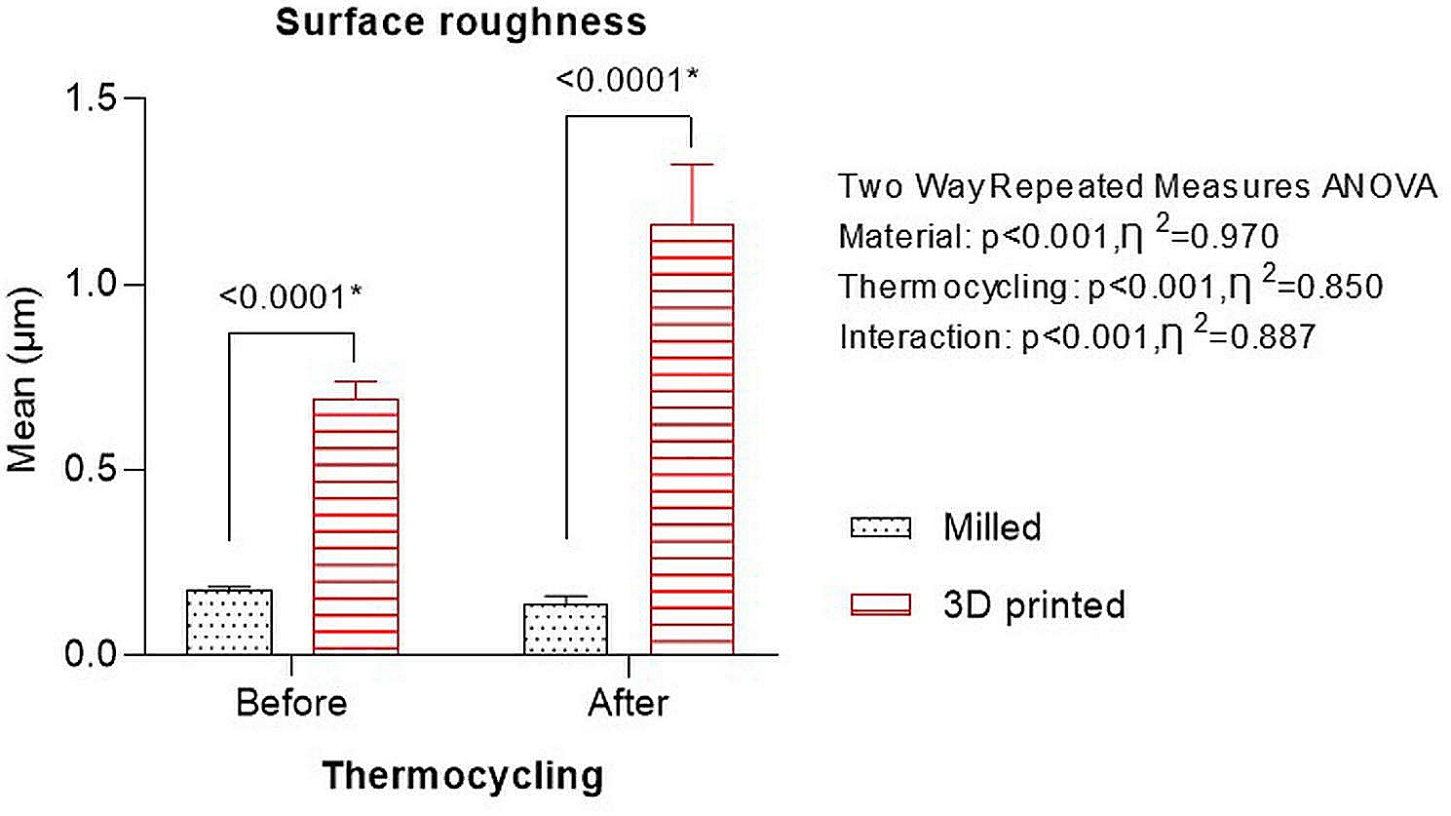
Graph showing surface roughness of CAD-CAM milled and 3D-printed denture base resins before and after thermocycling

The values of roughness of the surface of the 3D-printed resin were greater compared to CAD-CAM milled group both prior to (0.69 ± 0.05 and 0.18 ± 0.01, respectively) and following thermocycling (1.16 ± 0.16 and 0.14 ± 0.02, respectively). The surface roughness of both groups significantly increased following thermocycling (Fig. 5). There was significant effect of denture base material (*P* < .001), thermocycling (*P* < .001), as well as their significant interaction (*P* < .001) on surface roughness as revealed by repeated two-way repeated ANOVA test.

**Fig. 6:**
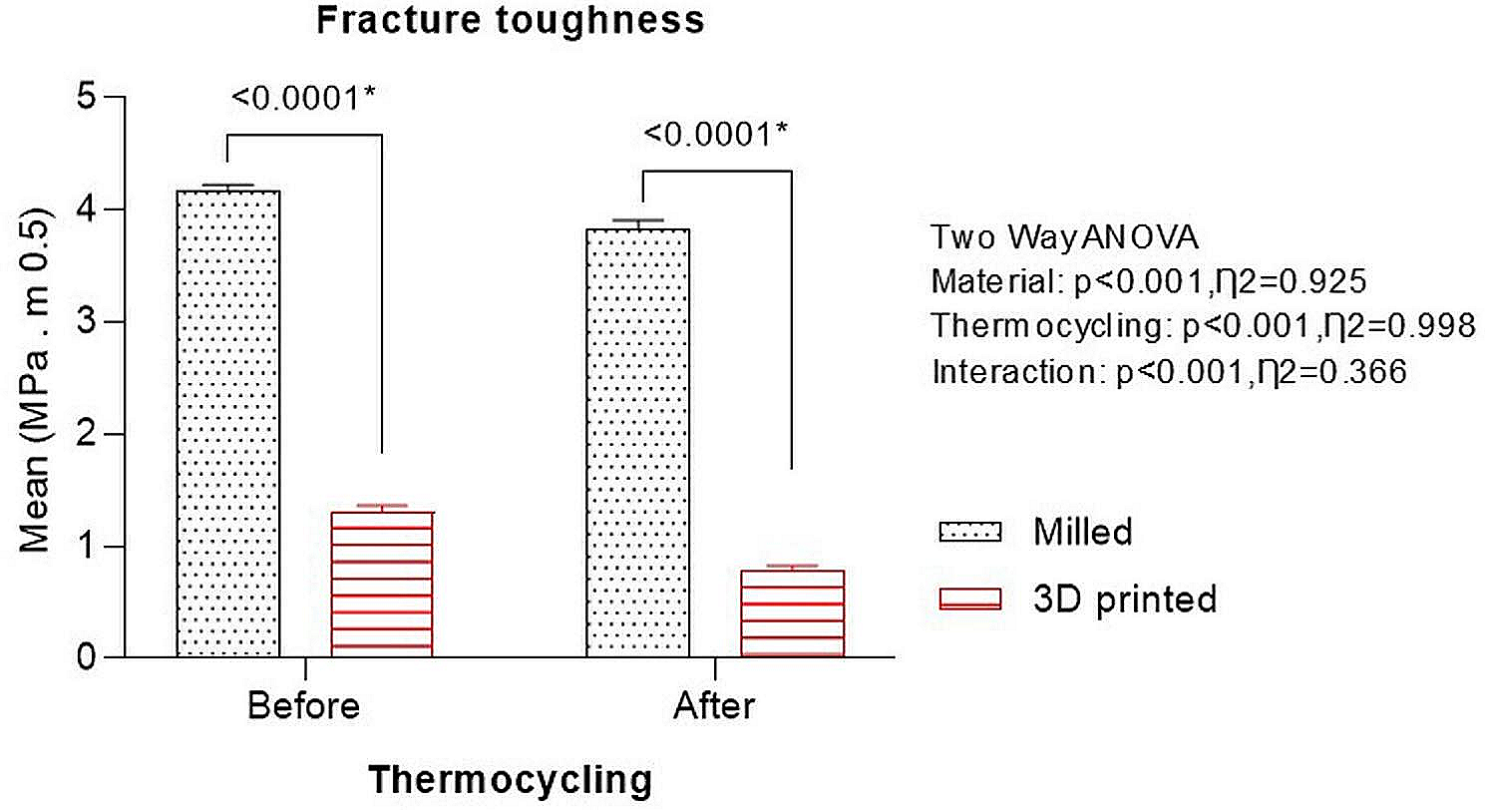
Graph showing fracture toughness of CAD-CAM milled and 3D-printed denture base resins before and after thermocycling

Fracture toughness values of CAD-CAM milled group were higher compared with 3D-printed group before (4.16 ± 0.06 and 1.30 ± 0.06, respectively) and after thermocycling (3.82 ± 0.08 and 0.78 ± 0.05, respectively). Thermocycling significantly decreased the fracture toughness of both groups (Fig. 6). There was significant effect of denture base material (*P* < .001), thermocycling (*P* < .001), and a significant effect of their interaction (*P* < .001) on fracture toughness as revealed by the two-way ANOVA test.

**Fig. 7:**
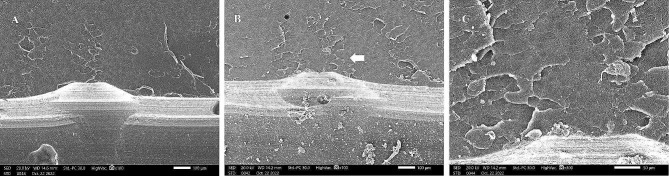
SEM images of fractured CAD-CAM milled denture base resin after fracture toughness test. A, Before thermocycling (×100). B, After thermocycling with white arrow pointing to jagged structure and small cracks (× 100). C, Higher magnification of B (× 300)

**Fig. 8:**
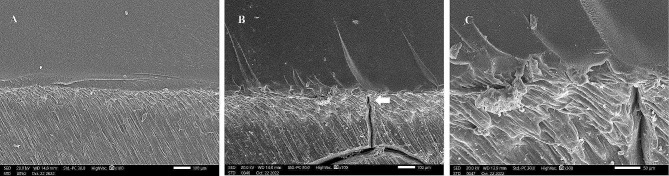
SEM images of fractured 3D-printed denture base resin denture base resin after fracture toughness test. A, Before thermocycling (×100). B, After thermocycling with white arrow pointing to jagged structure and small cracks (× 100). C, Higher magnification of B (× 300)

SEM images were taken for the internal fractured surfaces of specimens initiated by the pre-crack after the three-point bending test and revealed a compact structure in the CAD-CAM milled group with small irregular cracks and a jagged structure indicating slow crack propagation (Fig. 7). However, images of the 3D-printed group after thermocycling revealed a layered structure with sharp and wide cracks indicating fast crack propagation and delamination of layers (Fig. 8).

No difference was found in water sorption of CAD-CAM milled denture base prior to and following thermocycling (*P* = .154). However, water sorption of 3D-printed denture base resins decreased after thermocycling with a significant difference (*P* = .020). Water sorption of the 3D-printed group was greater before thermocycling with a significant difference (*P* = .003) However, after thermocycling the water sorption of both groups was similar (*P* = .562). Furthermore, the water solubility of both materials showed no significant difference (*P* = .759) (Table 2).

**Table 2:** Comparison of water sorption and solubility (µg/mm^3^) between the study groups

	Milled (*n* = 10)		3D-printed (*n* = 10)		*P* value
	Median (IQR)	Min - Max	Median (IQR)	Min – Max	
Wsp 84 h	3.98 (0.99)	3.98–7.96	11.94 (4.97)	3.98–19.89	0.003*
Wsp after thermocycling	0.00 (7.96)	-3.98–19.89	1.99 (12.93)	-11.94–11.94	0.562
*P* value	0.154		0.020*		
Wsl	-5.97 (23.87)	-15.92–11.94	-3.98 (11.94)	-15.92–3.98	0.759

*Statistically significant difference at *P* < .05

Table 3 shows the percent changes in values of Vickers hardness, surface roughness, fracture toughness, and water sorption and solubility after thermocycling in both groups. The percent change of hardness in the 3D-printed resin (-23.09 (14.72)) was higher than the milled resin (-11.29 (5.15)) with a statistically significant difference (*P* = .001). The percent changes of surface roughness and fracture toughness in the 3D-printed group were significantly greater than the milled group (*P* < .001). The percentage change in water sorption after thermocycling showed no significant difference between the 2 groups (*P* = .644).

**Table 3:** Percent change in all parameters after thermocycling

	Milled (*n* = 10)		3D printed (*n* = 10)		*P* value
	Median (IQR)	Min - Max	Median (IQR)	Min – Max	
Fracture Toughness	-8.13 (3.02)	-11.79 – -5.00	-39.22 (9.44)	-47.25 – -32.00	< 0.001*
Vickers Hardness	-11.29 (5.15)	-14.53–0.58	-23.09 (14.72)	-35.26 – -10.46	0.001*
Surface Roughness	-19.36 (14.10)	-45.21 – -6.70	73.08 (23.04)	34.90–93.14	< 0.001*
Wsp	-100.00 (200.00)	-200.00–150.00	-83.33 (175.00)	-200.00–100.00	0.644

*Statistically significant difference at *P* < .05

## Discussion

The goal of the present study was to test how thermocycling affected the surface topography and fracture toughness of denture base materials constructed by 3D printing and milling via CAD-CAM. The null hypothesis was partially rejected as results revealed that thermocycling influenced surface topography and fracture toughness and there was a difference between the two materials but there was no difference regarding water sorption and solubility.

Artificial thermocycling is used to mimic temperature changes in the oral cavity, imitating prolonged usage of dentures in the oral environment under temperature variations [35].

Hardness refers to the ability of a material to withstand localized plastic deformation caused by abrasion or mechanical indentation. Dentures with low surface hardness are susceptible to damage from mechanical brushing, which can lead to pigmentations and plaque retention, shortening their lifespan [3].

In the present study, Vickers hardness of both milled and 3D-printed resin decreased following thermal cycling, however, the milled group values continued to be higher than the 3D-printed group. Furthermore, in comparison to the milled group, the 3D-printed group’s percentage change in Vickers hardness was noticeably higher. These results are consistent with Gad et al. [24], who found that 3D-printed resins used for denture bases had lower hardness values after thermocycling. This could be due to water uptake, which is a phenomenon that depends on temperature. The water, which is absorbed serves as a plasticizing agent, causing inter-chain bond cleavage and subsequently deterioration of mechanical properties which is further increased due to heat that may cause delamination of 3D printed layers [49]. This can be attributed to the microstructure of 3D printed denture base resins due to the layered structure as seen in SEM images. This is not seen in milled denture base resins because of the compact microstructure due to pre-polymerization under heat and pressure.

The ability of resin materials to absorb water is a process that is controlled by diffusion that happens either when it enters spaces like micro-voids or when it interacts with certain molecules [40]. The interaction is dependent on resin polarity, or the quantity of polar sites open to hydrogen bonding with water [27]. Residual monomer may elute, and internal stresses are released with time causing cracks to form with time which may influence the characteristics of the denture base resin [26]. Since they reflect the material resistance to the surrounding oral fluids, water sorption and solubility are important when evaluating the denture base durability [53].

In the present study, thermocycling did not affect water sorption of the CAD-CAM milled group, while the 3D-printed group displayed a different behavior and there was a significant difference after thermocycling. These results are similar to Berli et al. [53], who found that thermocycling had a significant effect on water sorption of 3D-printed materials but did not affect pressed and milled resins. However, our results are opposite to Gad et al. [25], who found that thermal cycling resulted in a significant increase of water sorption of the studied materials. This may be due to the different tested materials and decreased amount of residual monomer due to different post-printing polymerization parameters.

When comparing both groups, the water sorption of the 3D-printed group was significantly greater in comparison with the milled group before thermocycling. These findings are similar to Perea-Lowery et al. [48], Berli et al. [53], Hanno et al. [35], and Dimitrova et al. [36], who also found that 3D-printed resins exhibit higher water sorption, due to increased residual monomers.

Changes in intraoral temperature when fluids are present may cause acrylic resin to absorb water more quickly and weaken the qualities of the polymer [55]. Moreover, water molecules fill the spaces between polymer chains as they diffuse into the polymer mass, pushing them apart and influencing the mechanical properties in the process [55]. Furthermore, this phenomenon is made worse by the thermal stresses that higher temperatures represent, which further deteriorate the properties of polymers [56].

Surface roughness of denture base resins is very important because denture bases are in contact with oral tissues. Surface roughness values of both groups in the study increased after thermocycling. Denture base resin that was 3D-printed had rougher surfaces than resin that was CAD-CAM milled following thermocycling with a significant difference. The surface roughness values of milled resin did not exceed the clinically acceptable threshold of 0.2 μm [29]. These results are similar to that of Çakmak et al. [28], who found that roughness of CAD-CAM resins was lower than that of 3D-printed denture base materials after thermocycling. CAD-CAM dentures are fabricated from prepolymerized PMMA resin under heat and pressure which leads to less residual monomer and higher degree of polymerization with less voids and spaces. 3D-printed dentures, on the other hand, are printed one layer at a time and then polymerization follows printing. This may lead to a weak inter-layer bonding due to unreacted remaining monomers and thus a minimal level of polymerization with more voids and inter-layer spacings creating a rough surface [10]. Specimens were printed at an angle of 45º and this caused an increase in surface roughness values due to height of step edges and stepwise connection between layers [57].

The degree to which a material is resistant to the propagation of defects under an applied load is measured using its fracture toughness [58]. It represents the maximum amount of stress that a material can withstand before a crack starts and spreads throughout it [59]. One method that is frequently used is three-point bending of a sharply notched beam to ascertain the fracture toughness of denture bases [60]. A denture base material can withstand cracking if it has a high fracture toughness [58].

In the present study, fracture toughness of both groups decreased after thermocycling, however, the percentage decrease in the 3D-printed group was 5 times greater than the milled group with a significant difference. These findings contrast with Mann et al. [39], who found that 3D-printed denture base resin had greater fracture toughness than milled resin. This may be because they tested specimens without thermocycling. SEM images confirmed fracture results with images showing wide and long fractures in 3D-printed resin, which is due to delamination of layers, facilitated by heat.

Limitations of this study include the use of milled and 3D-printed materials from single manufacturers, and the use of relatively low number of thermocycles. Future investigation is needed to study materials from various manufacturers and to test denture base materials under higher number of thermocycles and in different aging solutions.

## Conclusions

The following conclusions were reached based on the results of this in vitro study:

1. Vickers hardness and fracture toughness of CAD-CAM milled and 3D-printed denture base materials decreased after thermocycling but those of CAD-CAM milled were higher with a significant difference.
2. The roughness of the surfaces of both CAD-CAM milled and 3D-printed denture bases increased after thermocycling but those of CAD-CAM milled were lower with a significant difference.
3. Thermocycling did not have any effect on sorption of water and solubility of both denture base materials.

### Electronic supplementary material

Below is the link to the electronic supplementary material.

Supplementary Material 1

Supplementary Material 2

Supplementary Material 3

Supplementary Material 4

Supplementary Material 5

Supplementary Material 6

## Data Availability

The datasets used and/or analysed during the current study are available from the corresponding author on reasonable request.

## Abbreviations

3D: Three-Dimensional
CAD-CAM: Computer-aided Design, Computer-aided Manufacturing
SEM: Scanning Electron Microscope
CDs: Complete Dentures
PMMA: Polymethylmethacrylate
STL: Standard Tessellation Language
ISO: Organization for International Standardization
CNC: Computer Numeric Controlled
VHN: Vickers Hardness Number
