# Supplementary material for: Effect of thermocycling on surface topography and fracture toughness of milled and additively manufactured denture base materials: an in-vitro study
Source: BMC Oral Health. 2024 Feb 23;24:267. doi: 10.1186/s12903-024-03991-7 (PMC10885363; doi:10.1186/s12903-024-03991-7)
Supplement: Supplementary file 1 — Supplementary Material 1 [file 12903_2024_3991_MOESM1_ESM.docx]

Table 1. Vickers Hardness (HV) of the study groups before and after thermocycling

| Thermocycling | Milled  (n=10) | | 3D-printed  (n=10) | |
| --- | --- | --- | --- | --- |
|  | Mean ± SD | 95% CI | Mean ± SD | 95% CI |
| Before | 18.02 ± 0.67 | 17.54, 18.50 | 16.26 ± 0.79 | 15.69, 16.83 |
| After | 16.26 ± 0.79 | 15.69, 16.83 | 12.42 ± 1.30 | 11.49, 13.35 |
