# Supplementary material for: Effect of thermocycling on surface topography and fracture toughness of milled and additively manufactured denture base materials: an in-vitro study
Source: BMC Oral Health. 2024 Feb 23;24:267. doi: 10.1186/s12903-024-03991-7 (PMC10885363; doi:10.1186/s12903-024-03991-7)
Supplement: Supplementary file 2 — Supplementary Material 2 [file 12903_2024_3991_MOESM2_ESM.docx]

Table 2: TWO Way Repeated ANOVA assessing the effect of material and thermocycling on Vickers Hardness (HV)

| Variables | Df | Mean Square | F test | *P* value | Ƞ2 |
| --- | --- | --- | --- | --- | --- |
| Material | 1 | 78.400 | 81.215 | <.001 | 0.819 |
| Thermocycling | 1 | 78.400 | 106.715 | <.001 | 0.856 |
| Interaction | 1 | 10.816 | 14.722 | .001 | 0.450 |

*Statistically significant difference at *P*<.05. Ƞ2: Partial Eta Squared
