# Supplementary material for: Effect of thermocycling on surface topography and fracture toughness of milled and additively manufactured denture base materials: an in-vitro study
Source: BMC Oral Health. 2024 Feb 23;24:267. doi: 10.1186/s12903-024-03991-7 (PMC10885363; doi:10.1186/s12903-024-03991-7)
Supplement: Supplementary file 3 — Supplementary Material 3 [file 12903_2024_3991_MOESM3_ESM.docx]

Table 3: Surface roughness (µm) of the study groups before and after thermocycling

| Thermocycling | Milled  (n=10) | | 3D-printed  (n=10) | |
| --- | --- | --- | --- | --- |
|  | Mean ± SD | 95% CI | Mean ± SD | 95% CI |
| Before | 0.14 ± 0.02 | 0.13, 0.15 | 0.69 ± 0.05 | 0.66, 0.72 |
| After | 0.18 ± 0.01 | 0.17, 0.18 | 1.16 ± 0.16 | 1.04, 1.28 |
