# Supplementary material for: Effect of thermocycling on surface topography and fracture toughness of milled and additively manufactured denture base materials: an in-vitro study
Source: BMC Oral Health. 2024 Feb 23;24:267. doi: 10.1186/s12903-024-03991-7 (PMC10885363; doi:10.1186/s12903-024-03991-7)
Supplement: Supplementary file 4 — Supplementary Material 4 [file 12903_2024_3991_MOESM4_ESM.docx]

Table 4: TWO Way Repeated ANOVA assessing the effect of material and thermocycling on surface roughness

| Variables | Df | Mean Square | F test | *P* value | Ƞ^2^ |
| --- | --- | --- | --- | --- | --- |
| Material | 1 | 5.882 | 589.502 | <.001* | 0.970 |
| Thermocycling | 1 | 0.462 | 101.893 | <.001* | 0.850 |
| Interaction | 1 | 0.641 | 141.439 | <.001* | 0.887 |

*Statistically significant difference at *P*<.05. Ƞ^2^: Partial Eta Squared
