# Supplementary material for: Effect of thermocycling on surface topography and fracture toughness of milled and additively manufactured denture base materials: an in-vitro study
Source: BMC Oral Health. 2024 Feb 23;24:267. doi: 10.1186/s12903-024-03991-7 (PMC10885363; doi:10.1186/s12903-024-03991-7)
Supplement: Supplementary file 5 — Supplementary Material 5 [file 12903_2024_3991_MOESM5_ESM.docx]

Table 5: Fracture toughness (MPa. m ^0.5^) of the study groups before and after thermocycling

| Thermocycling | Milled  (n=10) | | 3D-printed  (n=10) | |
| --- | --- | --- | --- | --- |
|  | Mean ± SD | 95% CI | Mean ± SD | 95% CI |
| Before | 4.16 ± 0.06 | 4.12, 4.20 | 1.30 ± 0.06 | 1.26, 1.34 |
| After | 3.82 ± 0.08 | 3.76, 3.88 | 0.78 ± 0.05 | 0.74, 0.81 |
