# Supplementary material for: Effect of thermocycling on surface topography and fracture toughness of milled and additively manufactured denture base materials: an in-vitro study
Source: BMC Oral Health. 2024 Feb 23;24:267. doi: 10.1186/s12903-024-03991-7 (PMC10885363; doi:10.1186/s12903-024-03991-7)
Supplement: Supplementary file 6 — Supplementary Material 6 [file 12903_2024_3991_MOESM6_ESM.docx]

Table 6: TWO Way ANOVA assessing the effect of material and thermocycling on fracture toughness.

| Variables | df | Mean Square | F test | *P* value | Ƞ^2^ |
| --- | --- | --- | --- | --- | --- |
| Material | 1 | 1.855 | 445.701 | <.001 | 0.925 |
| Thermocycling | 1 | 87.174 | 20944.070 | <.001 | 0.998 |
| Interaction | 1 | 0.086 | 20.772 | <.001 | 0.366 |

*Statistically significant difference at *P*<.05. Ƞ^2^: Partial Eta Squared

Model summary: Adjusted R^2^: 0.998, *P* value<.001, Ƞ^2^: 0.998
